# Supplementary material for: VGRCOT: a one-tube visual detection method for group B Streptococcus combining RPA and CRISPR/Cas12a for point-of-care testing in reproductive health
Source: Microbiol Spectr. 2025 Aug 14;13(10):e01395-25. doi: 10.1128/spectrum.01395-25 (PMC12502718; doi:10.1128/spectrum.01395-25)
Supplement: Supplemental material — Fig. S1 to S5; Tables S1 and S2. [file spectrum.01395-25-s0001.docx]

**VGRCOT:** **a one-tube visual detection method for group B *Streptococcus* combining RPA and CRISPR/Cas12a for point-of-care testing in reproductive health**

**Caixia Ji^1†^,** **Liqiang Ru^2,3†^, Tiao Han****^4^, Gang Mai^5🖂^ Laibao Zheng^4🖂^, Yayun Jiang^5🖂^**

^†^ These authors contributed equally: Caixia Ji and Liqiang Ru.

**^🖂^** Corresponding authors: [maigang68@hotmail.com](mailto:maigang68@hotmail.com) (Gang Mai), zhenglaibao@wmu.edu.cn (Laibao Zheng), and dyjiangyayun@163.com (Yayun Jiang)


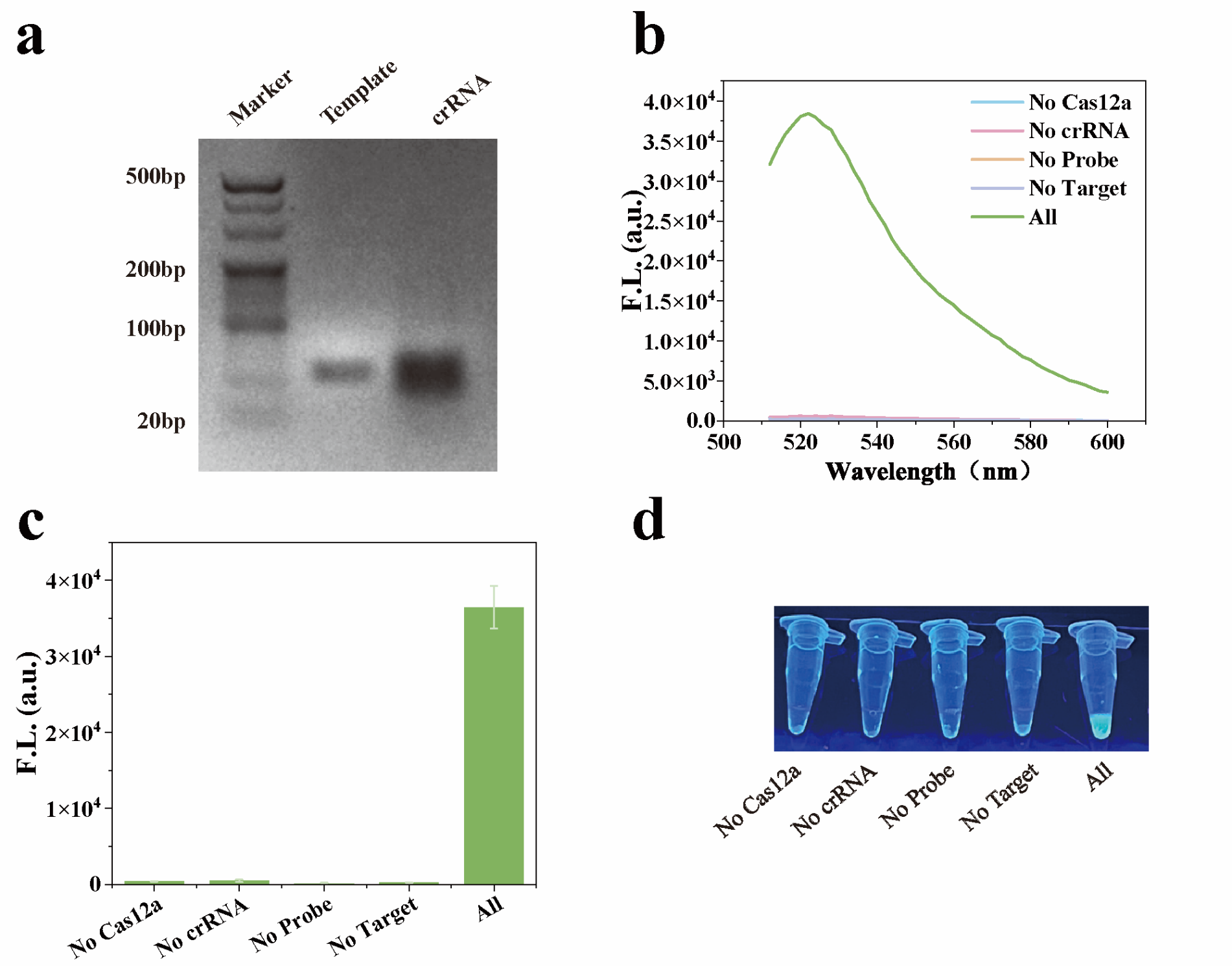


**Figure S1** Validation of the crRNA for CRISPR/Cas12a assay. (a) Analysis of the transcribed crRNA by 4% agarose gel electrophoresis. (b) Fluorescence spectra of CRISPR/Cas12a cleavage ssDNA-Probe in different reaction conditions. (c) A histogram of fluorescence intensity at 522 nm demonstrates the feasibility of CRISPR/Cas12a cleavage ssDNA-Probe. (d) A representative photograph of each group was taken under UV light.


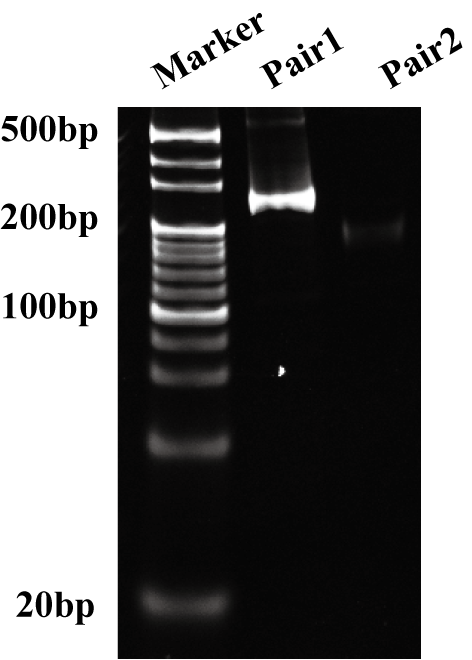


**Figure S2** PAGE analysis of amplification of the *cfb* gene by RPA with different primer sets.


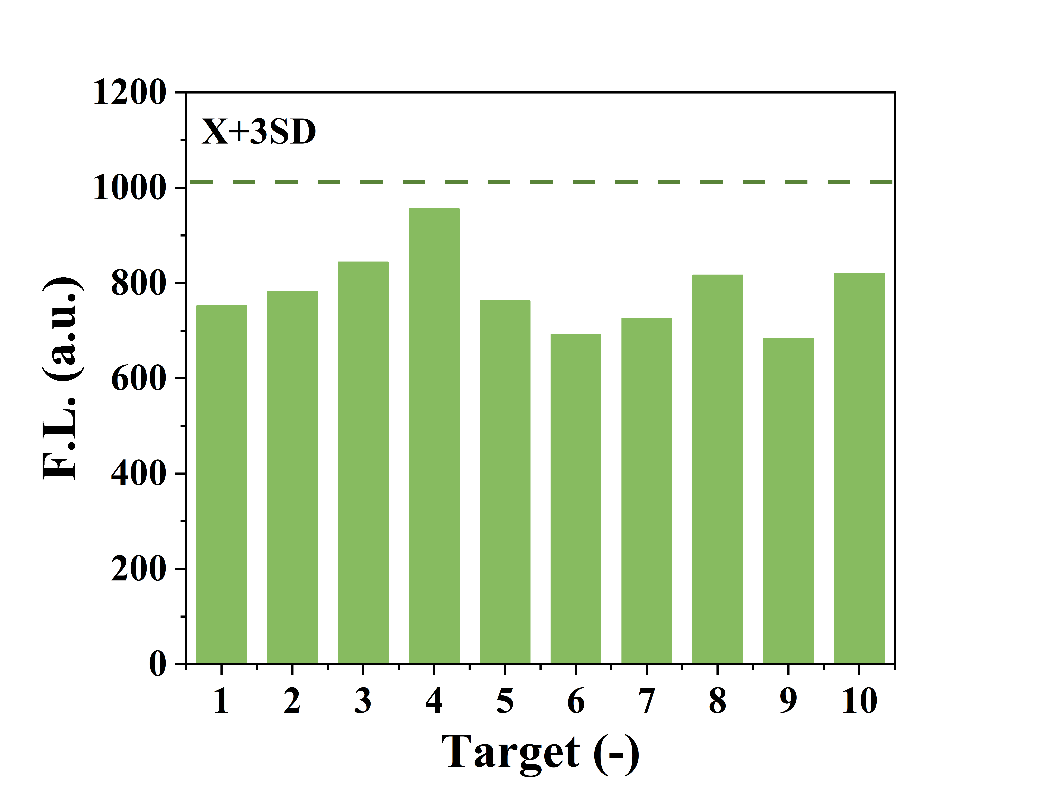


**Figure S3** Calculate the cut-off value for fluorescent outputs using the VGRCOT method. X + 3SD calculates the cut-off value, X represents the average signal value of three replicates without using the target gene, and SD represents three standard deviations.


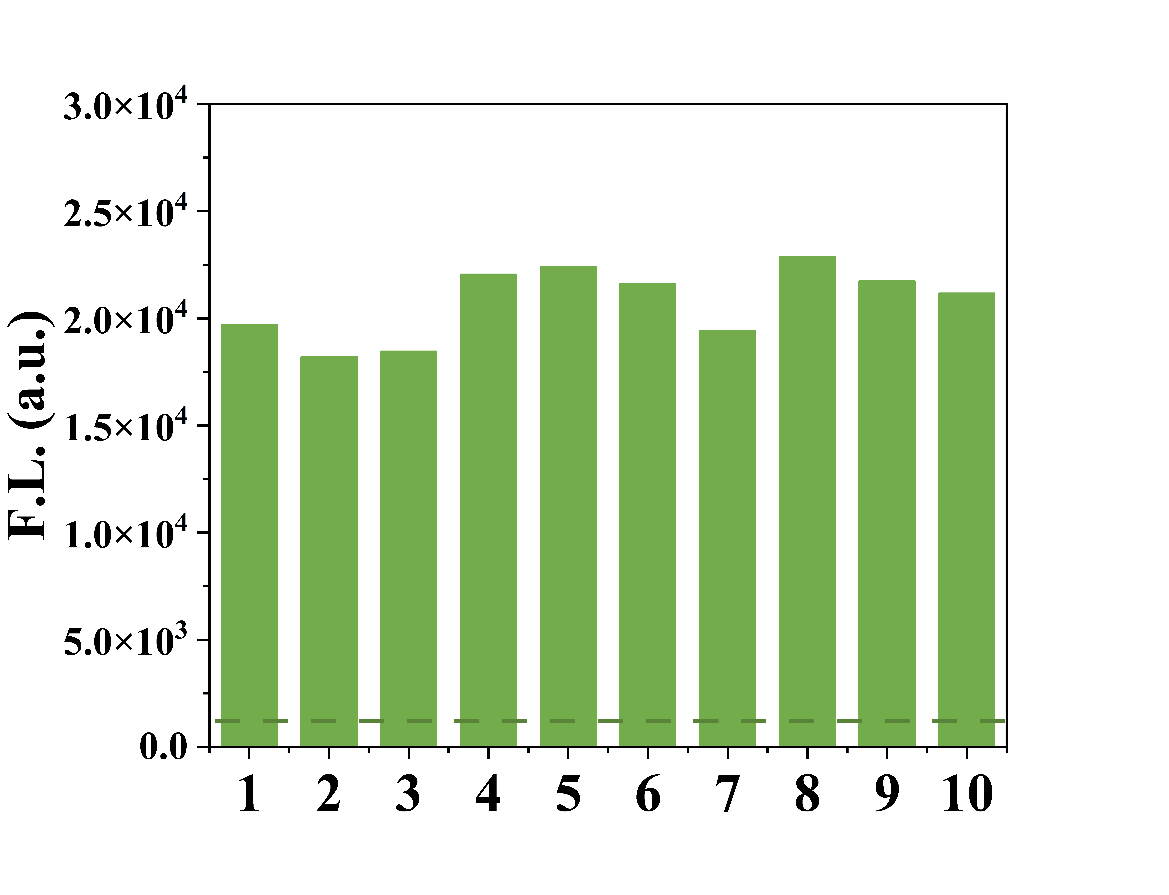


**Figure S4** The histogram of 10^1^ copies of *cfb* target genes detection.


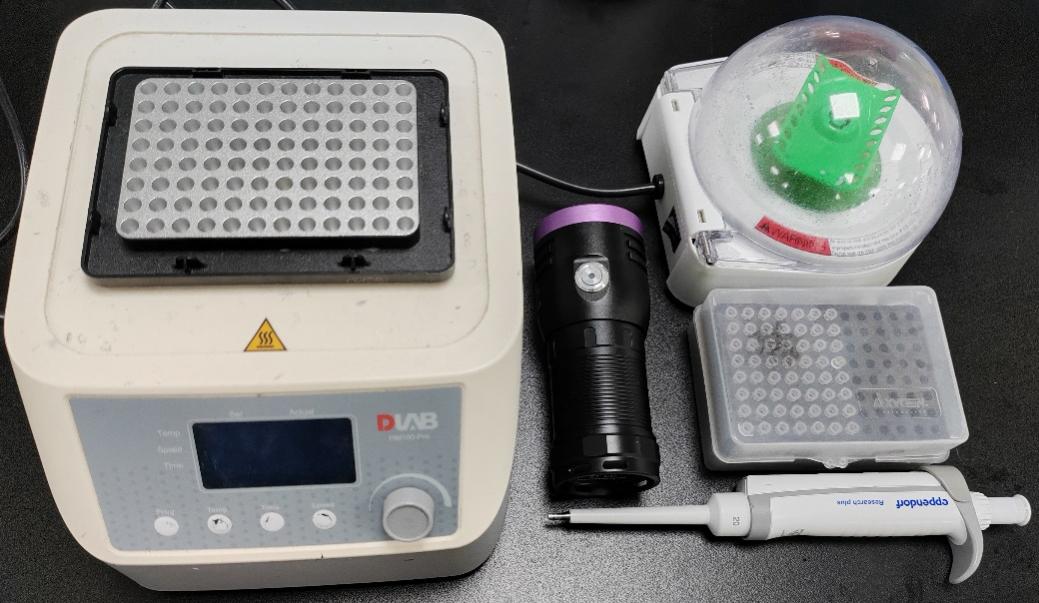


**Figure S5** The instruments required for the VGRCOT method.

**Table S1.** The sequences of oligonucleotides are used in this work.

| **Names** | **Sequences (5**'**-3**'**)** |
| --- | --- |
| *cfb*(F207) | CAGTAATAATCAAGCCCAGCAA |
| *cfb*(R207) | AAGGCTTCTACACGACTACCAA |
| *cfb*(F146) | AACCCTGAGACAGTTTAT |
| *cfb*(R146) | TTAGTTATCCCAAATCCC |
| *cfb* gene crRNA template | TCAAGATAGCATTCAGTTGAATCTACACTTAGTAGAAATTACCCTATAGTGAGTCGTATTA |
| crRNA | UAAUUUCUACUAAGUGUAGAUUCAACUGAAUGCUAUCUUGA |
| Probe | FAM-TTATT-BHQ1 |
| *cfb* gene | ATGAACGTTAAACATATGATGTATCTATCTGGAACTCTAGTGGCTGGTGCATTGTTATTTTCACCAGCTGTATTAGAAGTACATGCTGATCAAGTGACAACTCCACAAGTGGTAAATCATGTAAACAGTAATAATCAAGCCCAGCAAATGGCTCAAAAGCTTGATCAAGATAGCATTCAGTTGAGAAATATCAAAGATAATGTTCAGGGAACAGATTATGAAAAAACGGTTAATGAGGCTATTACTAGTGTTGAAAAATTAAAGACTTCATTGCGTGCCAACCCTGAGACAGTTTATGATTTGAATTCTATTGGTAGTCGTGTAGAAGCCTTAACAGATGTGATTGAAGCAATCACTTTTTCAACTCAACATTTAGCAAATAAGGTTAGTCAAGCAAATATTGATATGGGATTTGGGATAACTAAGCTGGTTATTCGCATTTTAGATCCATTTGCTTCAGTTGATTCAATTAAAGCTCAAGTTAACGATGTAAAGGCATTAGAACAAAAGGTTTTAACTTATCCTGATTTAAAACCAACTGATAGAGCTACAATCTATACAAAATCAAAACTTGATAAGGAAATCTGGAATACACGCTTTACTAGAGATAAAAAAGTACTTAACGTCAAAGAATTTAAAGTTTACAATACTTTAAATAAAGCAATCACACATGCTGTTGGAGTTCAGTTGAATCCAAATGTTACGGTACAACAAGTTGATCAAGAGATTGTAACATTACAAGCAGCACTTCAAACAGCATTAAAATAA |

**Table S2**. Validation of the VGRCOT repeatability experiments for 10^1^ copies of the *cfb* target genes

|  | 1 | 2 | 3 | 4 | 5 | 6 | 7 | 8 | 9 | 10 |
| --- | --- | --- | --- | --- | --- | --- | --- | --- | --- | --- |
| F.L. | 19708 | 18208 | 18460 | 22049 | 22404 | 21625 | 19422 | 22875 | 21740 | 21172 |
| SD | 1680.4 | | | | | | | | | |
| mean | 20766.3 | | | | | | | | | |
| CV% | 8% | | | | | | | | | |
